# Antibodies Capable of Enhancing SARS-CoV-2 Infection Can Circulate in Patients with Severe COVID-19

**DOI:** 10.3390/ijms241310799

**Published:** 2023-06-28

**Authors:** Andrey Matveev, Oleg Pyankov, Yana Khlusevich, Olga Tyazhelkova, Lyudmila Emelyanova, Anna Timofeeva, Andrey Shipovalov, Anton Chechushkov, Natalia Zaitseva, Gleb Kudrov, Gaukhar Yusubalieva, Saule Yussubaliyeva, Oxana Zhukova, Vladimir Baklaushev, Sergey Sedykh, Galina Lifshits, Artem Tikunov, Nina Tikunova

**Affiliations:** 1 Institute of Chemical Biology and Fundamental Medicine, Siberian Branch of the Russian Academy of Sciences, 630090 Novosibirsk, Russia; khlusevichjana@mail.ru (Y.K.); olia.golota@mail.ru (O.T.); mila.kuharenko@mail.ru (L.E.); anna.m.timofeeva@gmail.com (A.T.); achechushkov@gmail.com (A.C.); gl62@mail.ru (G.L.); arttik@ngs.ru (A.T.); tikunova@niboch.nsc.ru (N.T.); 2 State Research Center of Virology and Biotechnology “VECTOR”, Rospotrebnadzor, 630559 Koltsovo, Russia; pyankov@vector.nsc.ru (O.P.);; 3 Laboratory of Molecular Epidemiology and Biodiversity of Viruses, Research Institute of Virology, Federal Research Center of Fundamental and Translational Medicine, 630117 Novosibirsk, Russia; 4 Federal Research and Clinical Center for Specialized Types of Medical Care and Medical Technologies FMBA of Russia, 115682 Moscow, Russia; gaukhar@gaukhar.org (G.Y.); oksana.zhuk.82@inbox.ru (O.Z.); baklaushev.vp@fnkc-fmba.ru (V.B.); 5 Federal Center of Brain Research and Neurotechnologies, FMBA of Russia, 117513 Moscow, Russia; 6 Department of General Medical Practice with the Course of Evidence-Based Medicine, Astana Medical University, Nur-Sultan 010000, Kazakhstan; sm_yusubalieva@mail.ru; 7 Pulmonology Research Institute, FMBA of Russia, 115682 Moscow, Russia; 8 Engelhardt Institute of Molecular Biology, Russian Academy of Sciences, 119991 Moscow, Russia; 9 Faculty of Natural Sciences, Novosibirsk State University, 630090 Novosibirsk, Russia; sedyh@niboch.nsc.ru

**Keywords:** COVID-19, SARS-CoV-2, antibody-dependent enhancement, monoclonal antibody, virus neutralizing antibody, ADE

## Abstract

Antibody-dependent enhancement (ADE) has been shown previously for SARS-CoV-1, MERS-CoV, and SARS-CoV-2 infection in vitro. In this study, the first monoclonal antibody (mAb) that causes ADE in a SARS-CoV-2 in vivo model was identified. mAb RS2 against the SARS-CoV-2 S-protein was developed using hybridoma technology. mAb RS2 demonstrated sub-nanomolar affinity and ability to neutralize SARS-CoV-2 infection in vitro with IC_50_ 360 ng/mL. In an animal model of SARS-CoV-2 infection, the dose-dependent protective efficacy of mAb RS2 was revealed. However, in post-exposure prophylaxis, the administration of mAb RS2 led to an increase in the viral load in the respiratory tract of animals. Three groups of blood plasma were examined for antibodies competing with mAb RS2: (1) plasmas from vaccinated donors without COVID-19; (2) plasmas from volunteers with mild symptoms of COVID-19; (3) plasmas from patients with severe COVID-19. It was demonstrated that antibodies competing with mAb RS2 were significantly more often recorded in sera from volunteers with severe COVID-19. The results demonstrated for the first time that in animals, SARS-CoV-2 can induce antibody/antibodies that can elicit ADE. Moreover, in the sera of patients with severe COVID-19, there are antibodies competing for the binding of an epitope that is recognized by the ADE-eliciting mAb.

## 1. Introduction

Severe acute respiratory syndrome coronavirus-2 (SARS-CoV-2) is the causative agent of coronavirus disease 2019 (COVID-19) and, to date, according to the World Health Organization, more than 765 million people have been infected and more than 6.9 million people have died [1]. The SARS-CoV-2 virion consists of four structural proteins: spike protein (S), envelope protein (E), membrane protein (M), and nucleocapsid protein (N) [2]. S-protein contains two subunits, S1 and S2, and is located as a homotrimer on the outer membrane of the SARS-CoV-2 virion [3]. To fuse with the cell wall, the S-protein must switch to an open conformation. After that, the receptor-binding domain of the S1 subunit (RBD) becomes capable of binding with the ACE2 [4]. The use of blockers of RBD interaction with ACE2, such as antibodies, nanobodies, aptamers, and peptides, leads to a decrease In the infectivity of SARS-CoV-2 [5,6,7,8,9,10].

Vaccines against SARS-CoV-2 are the most effective agents for preventing COVID-19. Several SARS-CoV-2 vaccines developed on various platforms have been evaluated in clinical trials [11,12,13,14,15]. Although the use of SARS-CoV-2 vaccines has been demonstrated to be highly effective in infection control of COVID-19, the development of therapeutics is also required. One promising class of prophylactic and therapeutic agents against SARS-CoV-2 infection is drugs based on potent virus-neutralizing monoclonal antibodies (mAbs) [16,17,18,19].

Investigating the mechanisms of SARS-CoV-2 neutralization by antibodies is crucial for the development of effective and safe therapeutic agents. However, the antibody-mediated enhancement of infection (ADE) has been recorded for several viral infections, causing by Dengue virus, Zika virus, respiratory syncytial virus, SARS-CoV-1, Middle East respiratory syndrome coronavirus (MERS-CoV), and SARS-CoV-2 [20,21,22,23,24,25]. For this reason, it is necessary to investigate whether antibodies capable of ADEs emerge after SARS-CoV-2 infection or vaccination and whether such antibodies facilitate pathogenesis of COVID-19.

In this study, novel mouse monoclonal antibody (mAb) RS2 against the S-protein of SARS-CoV-2 was selected and investigated. It was shown that mAb RS2 has sub-nanomolar affinity and is capable of neutralizing SARS-CoV-2 infection in vitro with IC_50_ 360 ng/mL. In addition, mAb RS2 demonstrated dose-depended efficacy in pre-exposure prophylaxis in a hamster model of SARS-CoV-2 infection. Conversely, in post-exposure prophylaxis, the administration of mAb RS2 led to an increase in the viral load in the respiratory tracts of model animals. Using competitive ELISA, three groups of blood plasma were examined for antibodies competing with mAb RS2: (1) plasmas from vaccinated donors without COVID-19; (2) plasmas from volunteers with mild symptoms of COVID-19; (3) plasmas from patients from the intensive care unit (ICU) with a severe form of COVID-19. The levels of competing antibodies were compared between the groups of plasma samples.

## 2. Results

### 2.1. mAb RS2 Selection, Its Neutralization Activity, and Affinity

To develop anti-S mAbs, mice were immunized four times with the recombinant S-protein, and twelve hybridomas producing mAbs against S-protein were selected. After purification, all anti-S-protein mAbs were assessed for neutralizing activity in an in vitro neutralization assay. The nCoV/Victoria/1/2020 SARS-CoV-2 was neutralized only by mAb RS2, whereas other selected mAbs were not able to neutralize live virus. The IC_50_ for mAb RS2 was shown to be 360 ng/mL. To evaluate the binding affinity of mAb RS2, an ELISA was used. The affinity constant was calculated as KD = (0.39 ± 0.05) × 10^−9^ M. Using an ELISA, it was shown that mAb RS2 recognizes recombinant RBD at a concentration of 10 µg/mL or higher. This concentration is 500 times higher than for S-protein. Notably, unlike several other selected anti-S-protein mAbs, mAb RS2 did not reveal the recombinant S-protein that was used for mice immunization by Western blot analysis.

### 2.2. Epitope Mapping

For the epitope mapping, the phage display method was applied using combinatorial peptide phage libraries PhD-12 and PhD-C7C (New England Biolabs, Rowley, MA, USA). The PhD-12 phage library is a population of filamentous bacteriophages, each of which carries an insertion of a random peptide from 12 aa within the minor surface protein p3; phage library PhD-C7C contains a population of M13 phages, each carrying a random peptide of 7 aa flanked by cysteine residues, which leads to the formation of a CxxxxxxxC loop on the p3 protein surface. Before each round of biopanning, both libraries were independently depleted by incubation with non-specific mAb 3G11, having the same IgG1/kappa subclass as the mAb RS2, to eliminate phages binding to the constant domains of mouse antibodies.

The presence of an inserted peptide in the gene encoding the phage protein p3 was analyzed by PCR using primers pIII_93. The DNA fragments encoding the selected peptides were sequenced, and the deduced amino acid sequences of the peptide were compared with the amino acid sequence of the SARS-CoV-2 S-protein. As a result, two different peptide sequences were identified (Table S1). However, they did not match the S-protein sequence, meaning they were peptide-mimetics.

**Figure 1:**
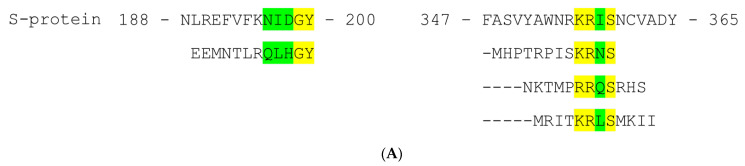

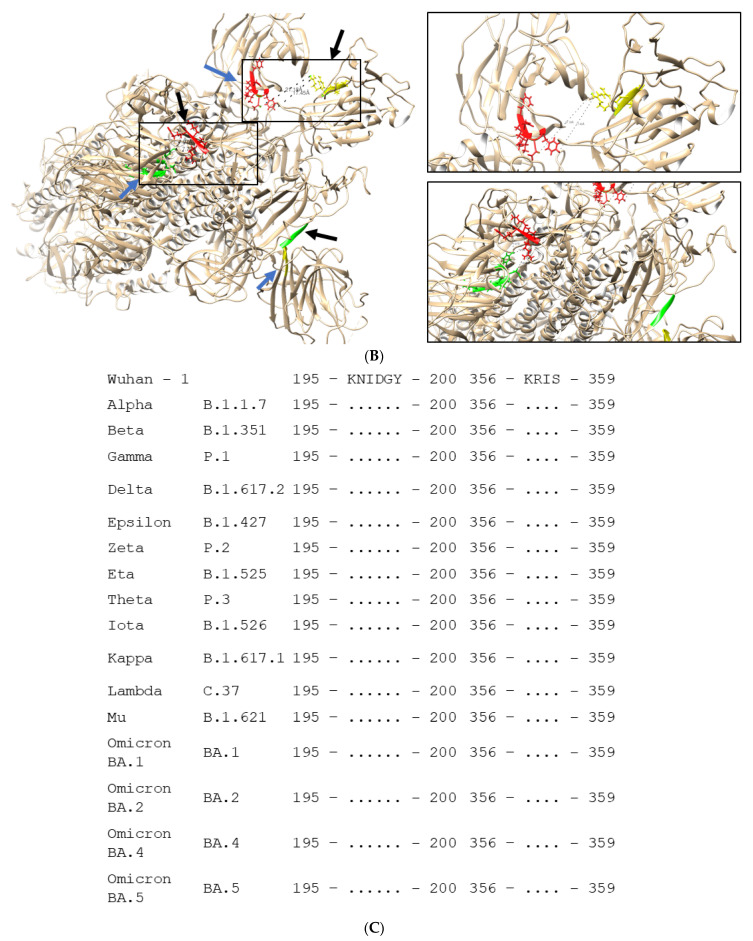
Epitope mapping of mAb RS2. (**A**) Results of phage display screening for peptides bound by mAb RS2. Alignment of S-protein of SARS-CoV-2 homolog fragments and peptides, selected from Ph.D.-12 phage display peptide library. (**B**) Ribbon representation of SARS-CoV-2 S-protein 3D structure (PDB 7VXE) with specified region 195–200 aa (blue arrow) and 356–359 aa (black arrow). The molecular coordinates for the structural analysis were derived from the Protein Data Bank and rendered using UCSF Chimera molecular visualizer, version 1.15. E—alignment of region 240–256 aa of TBEV glycoprotein E and peptides recognized by mAb FVN-32 selected from phage libraries. (**C**) Alignment of the identified fragments (195–200 aa and 356–359 aa) in different SARS-CoV-2 variants S-protein.

As for the PhD-12 library, 36 positive clones producing phages, which exposed peptides bound by mAb RS2, were selected. Sequencing indicated that selected peptides were mapped into two different regions of SARS-CoV-2 S-protein. Phages from 27 clones exposed the same peptide EEMNTLRQLHGY (Figure 1A). The selected peptide contains the motif ^195^BXAuGY^200^ (where B—basic a.a., X—polar a.a., A—aliphatic a.a., and u—any residue) found in the S1 domain of S-protein. Three more peptides were identified in the remaining clones (Figure 1A). They contain the common motif ^356^BRuS^359^ localized in the RBD domain. Despite the fact that these two sites are separated within the monomeric form of the S-protein, they can form a quaternary epitope in the S-protein trimer where these sites from different S-protein subunits are close (Figure 1B). Putative epitopes were highly conserved among all variants of SARS-CoV-2 (Figure 1C).

### 2.3. Pre- and Post-Exposure Administration of mAb RS2

Seven groups of hamsters were infected by an intranasal injection of 50 (infection doses) ID50 SARS-CoV-2 strain nCoV/Victoria/1/2020 SARS-CoV-2 suspended in 0.9% NaCl. One day before and one day after infection, six groups of hamsters were injected with mAb RS2 at doses of 20 mg/kg, 4 mg/kg, and 0.8 mg/kg, respectively. Two placebo groups of hamsters were treated with 0.9% NaCl and IgG1 isotype antibody [26], respectively.

**Figure 2:**
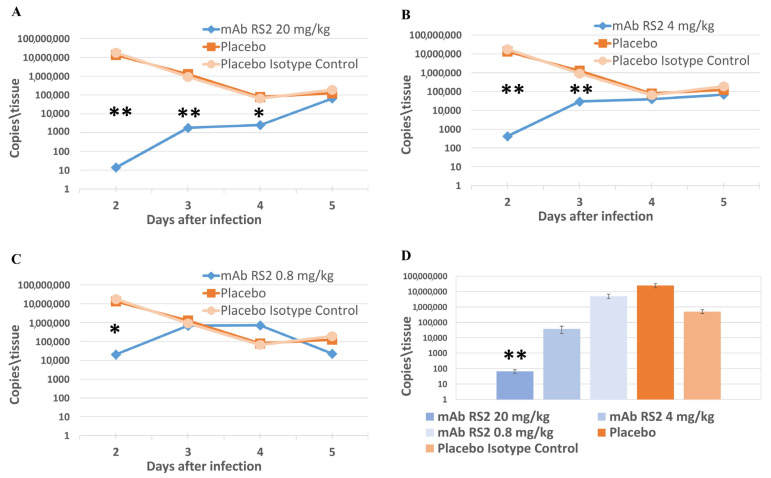
Pre-exposure mAb RS2 protection of hamsters infected with SARS-CoV-2. The viral loads in nasal fluids were measured using RT-PCR and were evaluated through a surrogate Ct indicator. (**A**)—dose of mAb RS2 20 mg/kg. (**B**)—dose of mAb RS2 4 mg/kg. (**C**)—dose of mAb RS2 0.8 mg/kg. (**D**)—The viral loads in lung homogenate at 120 h after infection. * *p* < 0.01; ** *p* < 0.001 (Mann–Whitney test).

Viral loads were assessed using RT-PCR via surrogate Ct value. The obtained results (Figure 2) indicated that the protective administration of mAb RS2 at doses of 20 mg/kg and 4 mg/kg led to a significant decrease in the viral load in nasal fluids (72 h after infection) and in lung homogenates (96 h after infection) compared to the placebo group (*p* < 0.01 by Mann–Whitney test) and a group of animals treated with 0.8 mg/kg mAb RS2 (*p* < 0.01 by Mann–Whitney test). The dose of 20 mg/kg substantially decreased the viral load in nasal fluids and lung tissue than the dose of 4 mg/kg. Notably, mAb RS2 was not effective at a dose of 0.8 mg/kg; however, the viral load in nasal fluids and lung homogenate at any day after pre-exposure prophylaxis in a group of mice administrated with mAb RS2 at a dose of 0.8 mg/kg was not higher than that in the placebo group.

**Figure 3:**
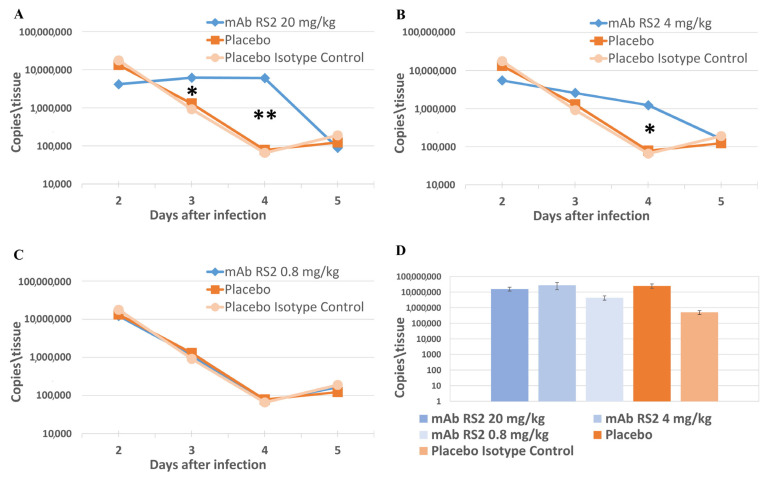
Post-exposure mAb RS2 protection of hamsters infected with SARS-CoV-2. The viral loads in nasal fluids were measured using RT-PCR and were evaluated through a surrogate Ct indicator. (**A**)—dose of mAb RS2 20 mg/kg. (**B**)—dose of mAb RS2 4 mg/kg. (**C**)—dose of mAb RS2 0.8 mg/kg. (**D**)—The viral loads in lung homogenate in 120 h after infection. * *p* < 0.01; ** *p* < 0.001 (Mann–Whitney test).

In contrast, post-exposure administration of mAb RS2 (Figure 3) led to the significantly higher viral load in nasal fluids 72 and 96 h after infection for a dose of 20 mg/kg and 96 h after infection for a dose of 4 mg/kg compared to the placebo group (*p* < 0.01 by Mann–Whitney test) and a group of animals treated with 0.8 mg/kg mAb RS2 (*p* < 0.01 by Mann–Whitney test).

Since a non-lethal model of SARS-CoV-2 was used, protective immunity to this virus was formed in the animal organisms and, normally, the virus disappeared. The obtained results indicated that in the used model, mAb RS2 postponed a decrease in the viral load in animals (Figure 3). As the time of mAb RS2 circulation in an animal is limited, the phenomenon of ADE was indicated at certain time points.

### 2.4. Competitive Analysis of Human Sera Antibodies with mAb RS2

To assess the association of antibodies against the RS2 epitope with the severity of COVID-19, a competitive ELISA with the mAb RS2 and antibodies from human blood was carried out. Three groups of blood plasma samples obtained were investigated: (1) plasma from donors, who never had COVID-19, but were vaccinated with Sputnik V (*n* = 33); (2) plasma from volunteers with mild COVID-19 (*n* = 106); and (3) from patients from ICU with severe COVID-19 (*n* = 94). Retrospective mortality of patients from ICU was more than 45%, whereas mortality among healthy donors and patients with mild COVID-19 was not recorded. All these blood plasmas were previously screened for IgG against SARS-CoV-2 proteins N and S [27]. In group 1, there were plasma samples containing antibodies against S-protein but not N-protein. All plasma samples from groups 2 and 3 contained antibodies against both SARS-CoV-2 proteins.

**Figure 4:**
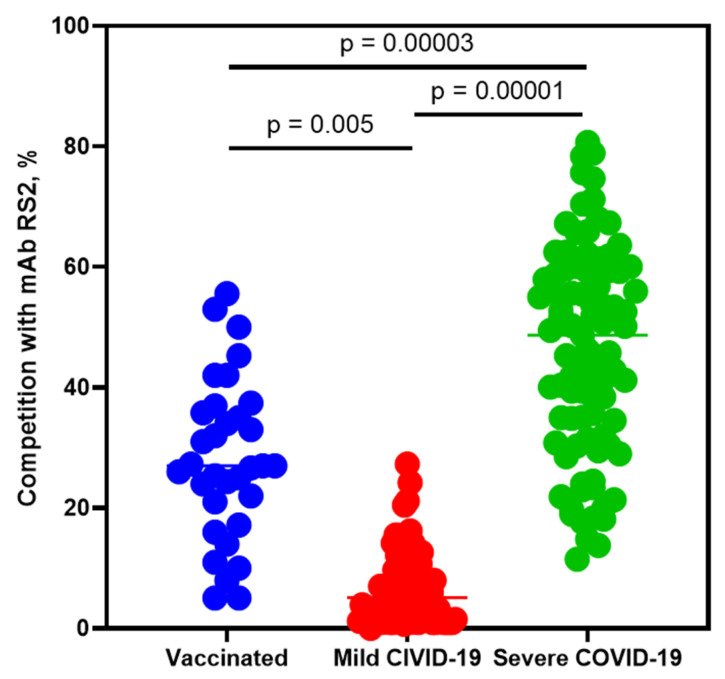
Data of competitive ELISA of sera with mAb RS2. The signal in the ELISA was taken as 100% competition when the antibody competed with itself. Blue—plasma from donors, who never had COVID-19, but were vaccinated with Sputnik V. Red—plasma from volunteers with mild COVID-19. Green—from patients with severe COVID-19 from the intensive care unit. Statistical analysis of the results was performed using one-way ANOVA.

A competitive ELISA was used for testing the above blood plasma samples for antibodies recognizing the mAb RS2 epitope. It was shown that the mean level of antibody competing with RS2 from blood plasma samples from groups 1 and 2 significantly differed from that of group 3 (28.0% and 6.7% versus 46.2%) and differed between group 1 and 2 (Figure 4). Three of thirty-three blood plasma samples (~9%) from group 1 contained antibodies competing with mAb RS2 by 49–55%, and no competing antibodies were found in the plasma samples from group 2 (Figure 4). The opposite situation was observed in plasmas from group 3: antibodies competing with mAb RS2 by 50% or more were detected in 48 samples of 94 (~51%), and 7 plasmas (~8%) had antibodies competing with mAb RS2 by 70% or more (Figure 4).

## 3. Discussion

One of the limitations in the development of vaccines and antibody preparations against new viral infections is the concern that antibodies could enhance the infection [28]. Previously, it was shown for MERS-CoV in vitro that antibodies against RBD of the virus can increase infection by penetrating into cells through the Fc receptor [29]. Unlike dengue infection, when ADE is observed in persons who recovered from dengue and then were infected with dengue virus of a different serotype, in the case of MERS-CoV, specific antibodies could increase infection when infected with the same strain [28]. It has been previously shown that in a non-lethal rabbit model of MERS infection, viral neutralizing antibodies were not formed in animals upon primary infection, and reinfection resulted in increased lung inflammation without an increase in viral RNA titer [29]. In addition, passive serum transfer from previously infected rabbits to naive rabbits was also associated with increased inflammation after MERS infection. The main reason for the enhancement of MERS infection in vivo was the increased activation of the complement system compared to the primary MERS infection [29]. Notably, ADE has been demonstrated for SARS-CoV-1 in in vivo experiments [23]. Anti-S antibodies produced after vaccination with inactivated SARS-CoV-1 caused hypercytokinemia and severe inflammation that trigged to fatal acute lung injury in the SARS-CoV-1 macaque model [23]. As for SARS-CoV-2, Fc-dependent ADE of infection were recorded in vitro using SARS-CoV-2 pseudovirus and FcγRIIB-expressing B cells for two human mAbs, MW01 and MW05 [30]. It has been shown that monovalent mAbs MW01 and MW05 against RBD completely lacked ADE activity in contrast to their bivalent equivalents [30]. Thus, a novel in vitro ADE mechanism has been demonstrated, in which an increase in FcγRIIB-mediated pseudovirus SARS-CoV-2 penetration into the target cell is associated with a bivalent interaction in the mAb/SARS-CoV-2 complex [30]. The effect of the ADE of convalescent serum samples in vitro has also been shown using cells expressing FcyR [31]. ADE manifested in a narrow range of human serum dilution and it was pronounced in average serum dilution, similar to that observed for dengue virus [32]. In addition, ADE was not associated with the neutralizing titer or the level of anti-RBD antibodies when testing serum samples taken from different patients [31]. In another study, the authors described convalescent sera exhibiting ADE and concluded that ADE is more likely to develop in elderly patients with severe and critical condition, longer hospital stays, and disease duration [33].

For some viruses (orthopoxviruses, tick-borne encephalitis virus), ADE, which was observed in vitro, has never been recorded in in vivo experiments and presumably does not contribute to the pathogenesis of viral infection [34,35]. So, it is important to investigate the antibody-mediated enhancement of infection (AME) in vivo.

It is believed that in most cases, ADE can be induced either with the use of a weakly neutralizing antibody or with sub-neutralizing concentrations of potent antibodies [25,36,37]. So, the formation of sub-neutralizing concentrations of antibodies is a normal phase of antibody response during infection [37]. In this study, we obtained a new high-affinity but weakly neutralizing mAb RS2 capable of enhancing SARS-CoV-2 infection in a non-lethal in vivo model. The administration of a different concentration of mAb RS2 after SARS-CoV-2 infection simulated different phases of the antibody response. As a result, the suboptimal mAb RS2 concentration that may contribute to amplification of the virus load in model animals was indicated.

It was demonstrated that antibodies competing with the mAb RS2 epitope are significantly more often circulating in the blood plasma of patients with severe COVID-19 than in patients with mild infection and healthy but vaccinated donors. Importantly, antibodies competing with mAb RS2 by 70% or more were detected only in the plasmas of patients with severe COVID-19. Thus, the presence of anti-RS2 antibodies in blood plasma is associated with the severity of COVID-19. So, antibodies that are potentially capable of enhancing SARS-CoV-2 infection can be elicited in humans.

We localized a putative epitope, recognized by mAb RS2 using phage display, which indicates that the epitope is quaternary. Western blot analysis and ELISA using RBD as an antigen confirmed our data. This epitope can be formed by two separate sites in the S-protein S1 domain ^(195^BXAuGY^200^) and RBD (^356^BBRuS^359^). Using in silico structural analysis, we determined probable distances between these two sites in the open and closed conformations of the RBD. In the closed conformation, the distance is approximately 7 Å, and mAb RS2 is able to efficiently bind the S-protein. When we determined the affinity constant, the obtained stabilized variant of the SARS-CoV-2 protein was used, in which the furin site was changed and RBD was always in the closed conformation. If RBD is in the open conformation, the distance between the sites increases three times, reaching 21 Å. Probably, mAb RS2 cannot bind the S-protein in this case because its quaternary epitope is violated. We hypothesize that a possible mechanism of virus neutralization by mAb RS2 is associated with the inhibition of the RBD transition from the closed to the open conformation required for virus entry into the cell. So, when one of the RBDs is pre-activated, mAb RS2 cannot prevent infection of the host cell with SARS-CoV-2.

Previously, two human mAbs, MW01 and MW05, also demonstrated Fc-dependent ADE of infection in vitro [30]. We assume that such antibodies do contribute to the development of more severe COVID-19. So, the possibility of the appearance of antibodies capable of ADE/AME of COVID-19 should be taken into account when developing new vaccines and therapeutics against the virus, which probably has been circulating in the human population for a long time.

## 4. Materials and Methods

### 4.1. Animals, Virus, Sera, and Cells

Strain SARS-CoV-2 nCoV/Victoria/1/2020 was received from the FSRC VB “Vector” repository. BSL-3 protocols were applied in experiments with live SARS-CoV-2.

Female mice (line BALB/c) and hamsters were acquired from the animal care facility of FSRC VB “Vector”. Animals were treated in a way described in recommendations for the use of laboratory animals used for experiments (EU Directive 2010/63/EU). Approval of the bioethics committee of FSRC VB “Vector” (Koltsovo, Novosibirsk Region, Russia) was received to perform all experiments with mice and hamsters.

CHO-S cells and expression plasmid pOptiCAG were obtained from the Collection of Extremophile Microorganisms and Type Cultures of ICBFM SB RAS.

This work was approved by the Local Ethics Committee of the Institute of Chemical Biology and Fundamental Medicine (Protocol Number 8 from 15 August 2021). According to the guidelines of the Helsinki ethics committee, the written consent of healthy donors and patients before hospitalization was obtained to present their plasma for scientific purposes.

Blood plasma samples were collected from three groups of volunteers: patients with severe COVID-19 from the intensive care unit, patients with mild COVID-19, and healthy vaccinated donors in Novosibirsk and Moscow between October 2020 and May 2021. Using an ELISA, all plasma samples were analyzed for the presence of antibodies against S- and N-proteins of SARS-CoV-2 [27]. The main clinical characteristics and outcome of patients with severe COVID-19 from ICU are provided in Table S2.

### 4.2. Antigens Production

The genes encoding the “stabilized” variant of the recombinant S-protein of SARS-CoV-2 and RDB of the S-protein (strain Wuhan-1 SARS-CoV-2 strain, GenBank: MN908947) were de novo synthesized by Genwiz, from Azenta Life Sciences company (Burlington, MA, USA), and inserted into the expression plasmid pOptiCAG. CHO-S cells were transfected by the resulting plasmids pOptiCAG-S and pOptiCAG-RBD using PEIpro (Polyplus, Illkirch, France) according to the manufacturer’s instructions. Recombinant RBD and S-protein were purified from culture media using agarose Ni-NTA resin (Qiagen, Hinden, Germany).

### 4.3. Mouse Immunization and mAbs Selection

To develop antibodies against S-protein of SARS-CoV-2, recombinant ectodomain of SARS-CoV-2 S-protein was used for immunization. To prepare the emulsion, 40 μg of recombinant S-protein in phosphate-buffered saline (PBS), pH 7.4, was suspended with an equal volume of Freund’s complete adjuvant. The prepared emulsion was used for the subcutaneous administration to 12–14-week-old female BALB/c mice (21–27 g). Each mouse was immunized with 40 μg of recombinant S-protein in phosphate-buffered saline emulsified with Freund’s incomplete adjuvant, two and four weeks after the first immunization. Final immunization was performed two weeks after the second infection and mice were immunized with S-protein in PBS without adjuvants. Three days after the final immunization, mouse splenocytes were obtained. Then, fusion of SP2/0 myeloma cells with obtained splenocytes was performed using PEG 2000 (Roche, Basel, Switzerland) in accordance with protocol described previously [26]. Hybridoma clones producing antibodies against SARS-CoV-2 S-protein were selected by anti-S-protein mAb titer in supernatants using ELISA. The limiting dilution method was applied two times to clone the hybridoma, producing mAb RS2 (IgG1 subclass with the kappa-type light chains).

### 4.4. Production, Purification, and Evaluation of the mAb RS2

Two 18-week-old BALB/c mice were injected intraperitoneally with hybridoma cells (2 × 10^6^) to produce mAb RS2. MabSelect protein A resin (GE Healthcare, Chicago, IL, USA) for affinity chromatography was applied to purify mAb RS2 from mouse ascites according to the mice IgG1 purification protocol. After purification, mAb RS2 was concentrated using Sartorius Vivaspin Turbo 4 50 kDa (Sartorius, Gottingem, Germany). Buffer (50 mM Tris-HCl, pH 7.5, 150 mM NaCl, 0.05% NaN_3_) was used to store mAb.

The affinity constant of mAb RS2 was determined using the protocol previously described by Levanov et al. [38]. The recombinant S-protein of SARS-CoV-2 was sorbed in 96-well microtiter plates (200 ng per well) at 4 °C overnight and then blocked with 5% skimmed milk for one hour at 37 °C. After that, the plates were washed with PBS. mAb RS2 was serially diluted in PBS containing 0.05% Tween-20 (PBST) and added to wells. Plates were incubated in a thermostat for one hour at 37 °C. Immune complexes were detected with an alkaline phosphatase-conjugated goat anti-mouse IgG (Sigma Aldrich, St. Louis, MO, USA) and visualized with para-nitrophenylphosphate. The sigmoid curves were drawn to view the interaction of OD_405_ versus lg(DM). DM represents the dilution multiples of antibody. Binding affinities were calculated according to the following equation:

$$OD_{405}\left(A_{0}\right)=N\times \left(A_{0}+\frac{b_{0}}{V}+K_{d}-\sqrt{{\left(A_{0}+\frac{b_{0}}{V}+K_{d}\right)}^{2}-4\times A_{0}\times \frac{b_{0}}{V}\;}\right),$$

where OD_405_ is the optical absorbance of solution in the microplate well, A_0_ is the total concentration of antibody in solution, b_0_ is the total concentration of the recombinant S-protein, K_d_ is the dissociation constant of antigen–antibody complex on the surface, V is the total volume of solution in the microplate well, and N is the normalization factor. Regression analysis was performed using the Origin 7.0 software.

For Western blot analysis, purified recombinant S-protein was fractionized by 12.5% SDS-PAGE and relocated to a nitrocellulose membrane. Skimmed milk, diluted in PBST, was used as a blocking agent. Then, the membrane was cut into strips and several strips were incubated with mAb RS2 (30 μg/mL) at 37 °C for one hour; incubation with anti-SARS-CoV-2 mice serum was used as a positive control. Goat anti-mice IgG conjugated with horseradish peroxidase (Sigma Aldrich, St. Louis, MO, USA) was used to reveal the antigen–antibody complexes which formed. 4-Chloro-1-naphthol (Sigma-Aldrich, St. Louis, MO, USA) was applied to visualize the complexes.

### 4.5. Assessment of Neutralization Activity In Vitro

Virus neutralizing activity of mAb RS2 was assessed in the inhibition of the cytopathic effect (CPE) as described earlier [39]. Consecutive five-fold dilutions of mAb RS2, starting at a concentration of 100 μg/mL, were mixed in a 1:1 ratio with a solution containing 100 cytopathic doses in 1 mL (TCID_50_) of SARS-CoV-2 virus strain nCoV/Victoria/1/2020 and incubated for 1 h at room temperature. Then, the mixture was poured onto a Vero E6 cells monolayer. Infected cells were cultivated for 4 days at 37 °C and then stained with 0.2% gentian violet solution. The presence of specific CPE was assessed visually by a microscopic examination of the cell monolayer. Antibody dilutions that completely prevented CPE in 50% of the wells were calculated according to the Reed–Munch method [40].

### 4.6. Animal Studies

A non-lethal model of hamster SARS-CoV-2 infection was described earlier [41]. Eight groups of six hamsters (six experimental groups and two control groups) were used. Each group contained three males and three females. The amount of viral RNA in nasal fluids and lung homogenates was assessed using real-time reverse transcription polymerase chain reaction (RT-PCR) through a surrogate Ct indicator (number of cycles). Animals were observed for five days and samples of nasal fluids were taken in 48, 72, 96, and 120 h after infection, as described previously [41]. Then, all animals were euthanized by dislocation of the cervical vertebrae and lung homogenates were obtained (in 120 h after infection) for RT-PCR [41].

### 4.7. Competitive ELISA

For a competitive ELISA, mAb RS2 was conjugated with biotin using an EZ-Link™ Sulfo-NHS-LC-Biotinylation Kit (Thermo Fischer Scientific, Waltham, MA, USA) according to the manufacturer instruction. S-protein was used as an antigen for competitive ELISA. The test sera were diluted 1:20. Biotin conjugated mAb RS2 were used at a concentration of 0.3 μg/mL. Streptavidin conjugated horseradish peroxidase (Sigma Aldrich, St. Louis, MO, USA) was used to reveal antigen–antibody complexes which formed. 4-Chloro-1-naphthol (Sigma-Aldrich, St. Louis, MO, USA) were applied to visualize the complexes. Reaction was stopped by adding 1 M NaCl. Optical density was measured on iMark plate reader at 450/600 nm. The level of competition was calculated according to the formula:

(1) $$C=\left(1-\;\frac{\mathrm{ODser}}{\mathrm{ODmin}}\right)\times 100$$

where C is the percentage of competition, ODser is the optical signal in the well with the tested serum, and ODmin is the optical signal in the well without serum.

### 4.8. Epitope Mapping

The phage display method was performed for epitope mapping using the phage display libraries PhD-C7C and PhD-12 (New England Biolabs, Ipswich, MA, USA). Specific peptides were selected as described in Matveev et al. [42]. Non-specific mAb 3G11, belonging to the IgG1 sub-class with a kappa-type constant region of light chains [26], was incubated with phage particles (10^11^) from each library. After depletion, phage particles were added to 96-well plates with sorbed RS2 (200 ng per well) in PBS. After incubation at 37 °C for 60 min, unbound phages were removed. The elution of specific phages was performed with 100 mkg/mL mAb RS2 and eluted phages were used for the next rounds of biopaning. Only 20 ng per well of RS2 was used in the following biopaning rounds. *E. coli* cells (ER2738) were transfected by bacteriophages eluted after the second round of biopaning. Individual plaques were used to obtain individual bacteriophages. The phages were sequenced and tested in an ELISA to bind RS2.

For indirect ELISA, 20 ng mAb RS2 was absorbed in 96-well plates. Unique selected phages were used at 10^10^ approximate concentrations. Bound phage particles exposing the peptide were detected with rabbit anti-M13-polyclonal antibodies. The immune complexes formed were detected by the conjugate of alkaline phosphatase and anti-rabbit antibodies, which were detected by para-nitrophenylphosphate (Roche, Basel, Switzerland). As a negative control, we used the detection of sorbed mAb RS2 with “wild-type” bacteriophage M13, which does not carry a peptide insertion on the surface of the protein p3.

To determine the aa sequences of the RS2-specific peptides selected from library, the appropriate gene fragments encoding these peptides were amplified by PCR using oli-gonucleotides M13_pIII_F (5′-CTCTGTAGCCGTTGCTAC-3′) and M13_PIII_96 (5′-CCCTCATAGTTAGCGTAACG-3′). Then, the fragments were sequenced using the BigDye Terminator v3.1 cycle sequencing kit and the 3500 DNA Analyzer (Applied Bio-systems, Foster City, CA, USA).

### 4.9. Statistics

Statistical analysis of the in vivo experiment was performed using the Mann–Whitney U-test. The results of the competitive ELISA were evaluated using a one-way ANOVA test. Statistical analysis was made with GraphPad Prism 9 software (Graphpad Software Inc., San Diego, CA, USA). The *p* value was 0.05 or less; the differences between the groups were considered to be significant.

## Data Availability

Not applicable.

## Supplementary Materials

The following supporting information can be downloaded at: https://www.mdpi.com/article/10.3390/ijms241310799/s1.
