# Supplementary material for: Antibodies Capable of Enhancing SARS-CoV-2 Infection Can Circulate in Patients with Severe COVID-19
Source: Int J Mol Sci. 2023 Jun 28;24(13):10799. doi: 10.3390/ijms241310799 (PMC10341398; doi:10.3390/ijms241310799)
Supplement: Supplementary file 1 [file ijms-24-10799-s001.zip › ijms-2443358-supplementary.pdf]

Supplementary information

Table S1. Phage display peptides, recognizing by mAb RS2.

Phage library	Peptide
Ph.D. 12	MHPTRPISKRNS
Ph.D. 12	NKTMPRRQSRHS
Ph.D. 12	MRITKRLSMKII
Ph.D. 12	EEMNTLRQLHGY
Ph.D. C7C	ACTRRQMNNC
Ph.D. C7C	ACSLHHVAGC

Table S2. Characteristics of the COVID-19 patients admitted to the ICU.

	Total (n = 94)
Age, y	62 (35 – 82)
Sex, male	47 (50)
BMI, kg/m2	28.1 (23.1 – 31.3)
Routine tests	
C-reactive protein, mg/L (N <5 mg/L)	153 (103 – 210)
Lymphocytes, 109/L (N 1.5–4.5 109/L)	0.9 (0.5 – 1.1)
Ferritin, μg/L (N 11–336 μg/L)	876 (375 – 1830)
IL-6, pg/mL (N <7 pg/mL)	93.5 (49.8 – 190)
Outcomes	
LOS ICU, d	13 (3 – 28)
Hospital stay, d	19 (5 – 39)
Nonsurvivors	45%

Values are median for numerical data and n (%) for categorical data. ICU, intensive care unit; LOS, length of stay; N, reference range;
